# Supplementary figures and images for: A genetic framework for RNAi inheritance in Caenorhabditis elegans
Source: EMBO Rep. 2025 Jul 7;26(16):4072–99. doi: 10.1038/s44319-025-00512-7 (PMC12373942; doi:10.1038/s44319-025-00512-7)

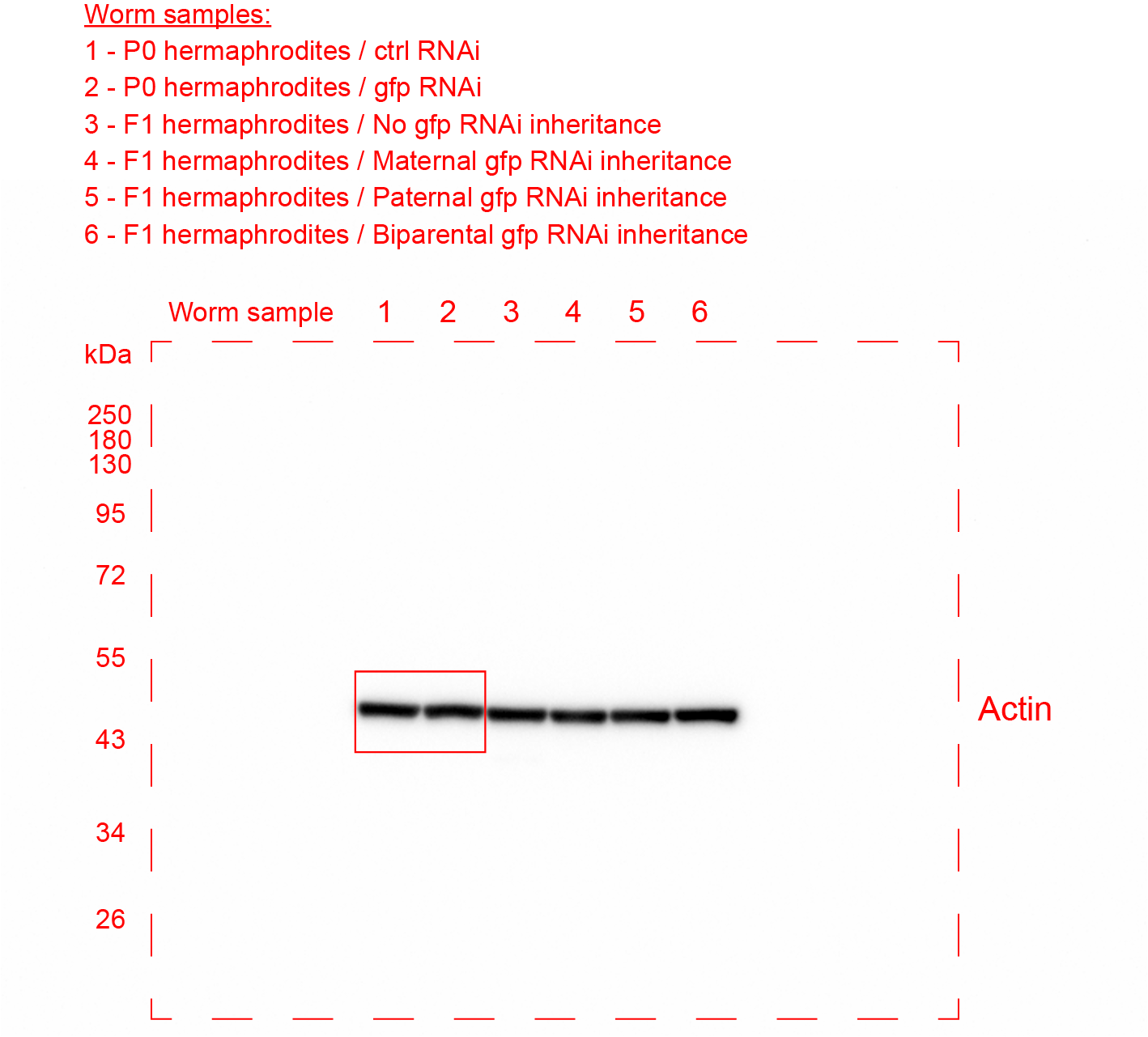

Supplement: Supplementary file 2 — Source data Fig. 1 [file 44319_2025_512_MOESM2_ESM.zip › Figure_1/1D/Western_Blot_anti-actin_P0_hermaphrodite.png]

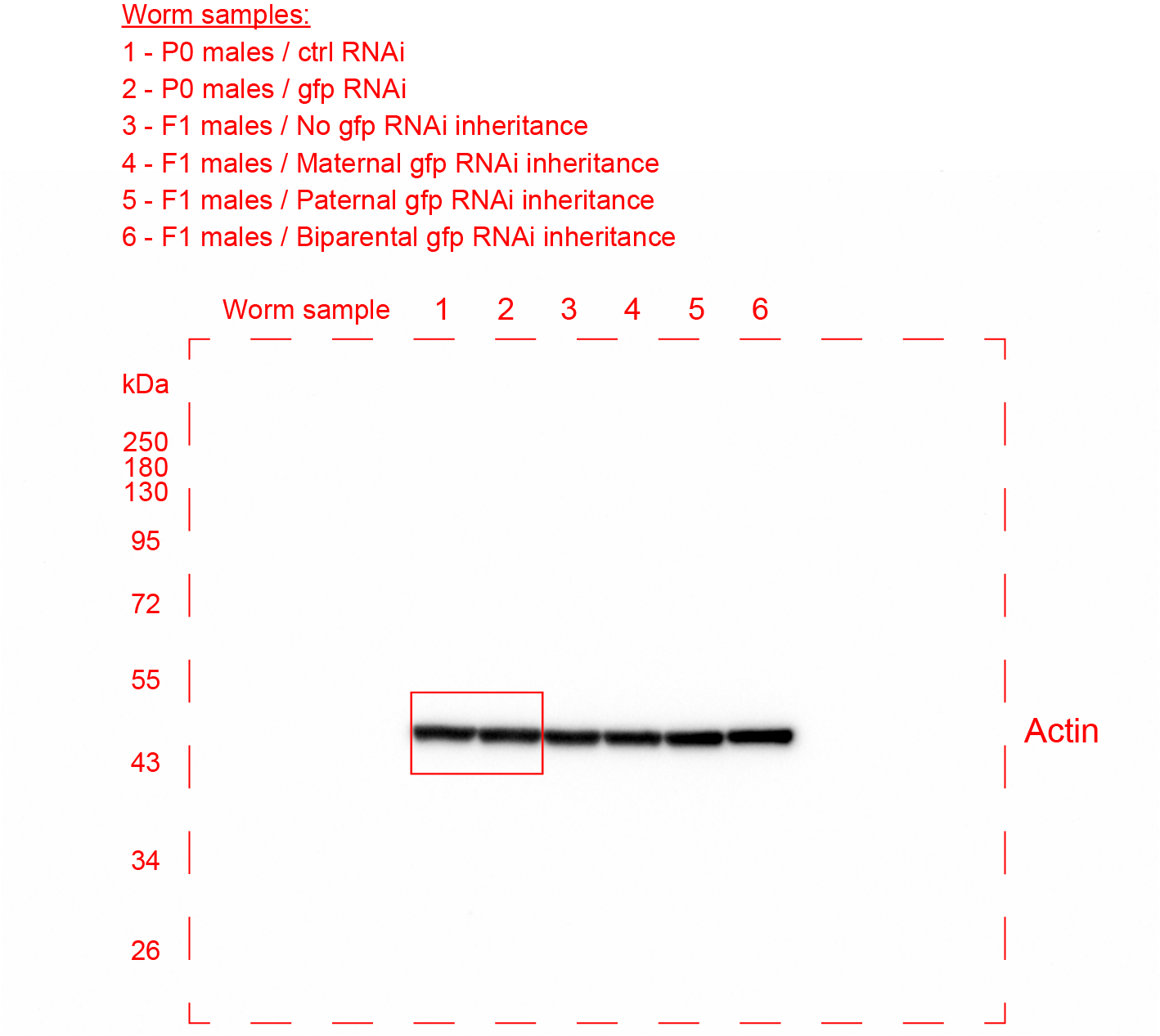

Supplement: Supplementary file 2 — Source data Fig. 1 [file 44319_2025_512_MOESM2_ESM.zip › Figure_1/1D/Western_Blot_anti-actin_P0_male.png]

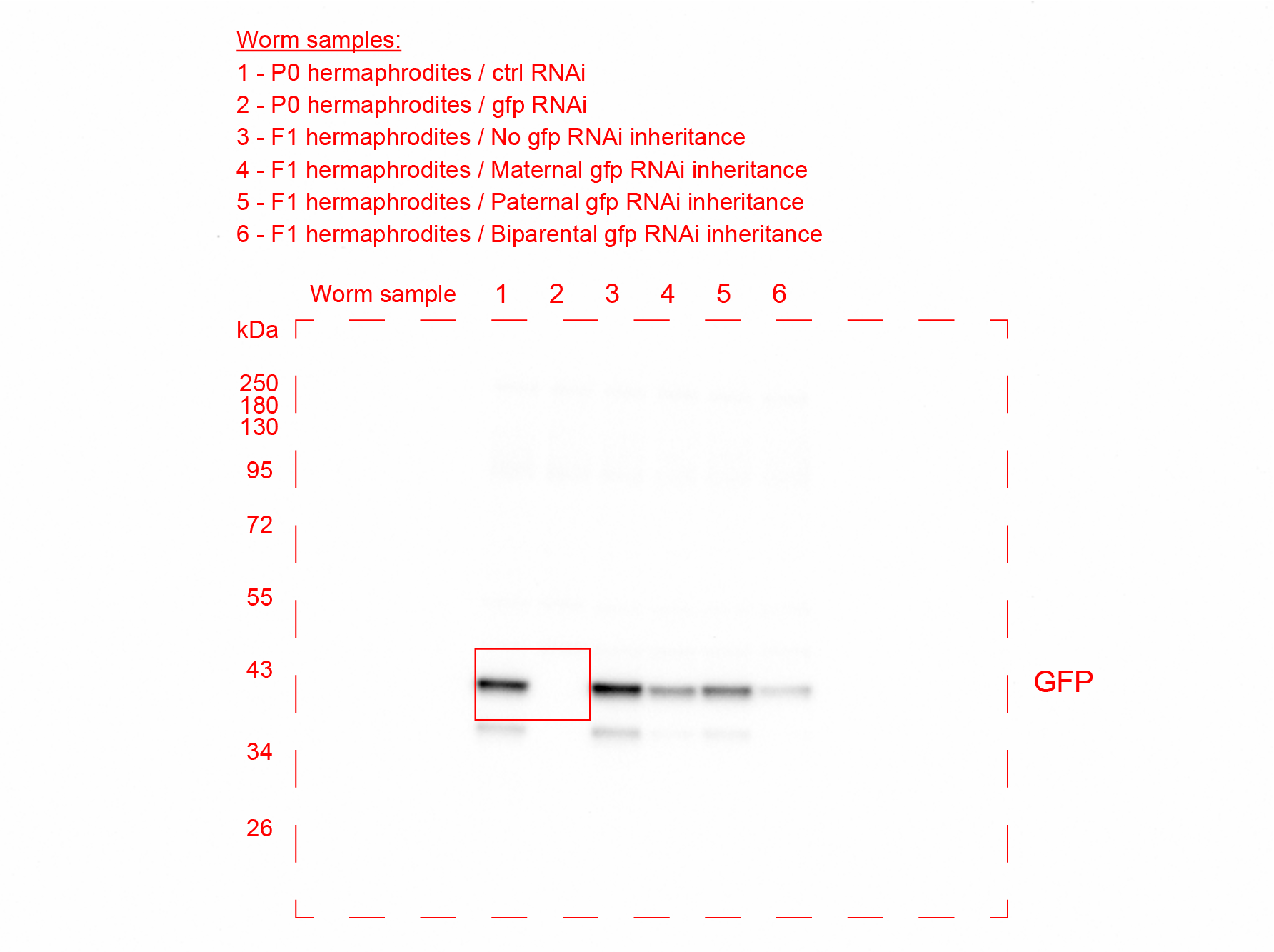

Supplement: Supplementary file 2 — Source data Fig. 1 [file 44319_2025_512_MOESM2_ESM.zip › Figure_1/1D/Western_Blot_anti-GFP_P0_hermaphrodite.png]

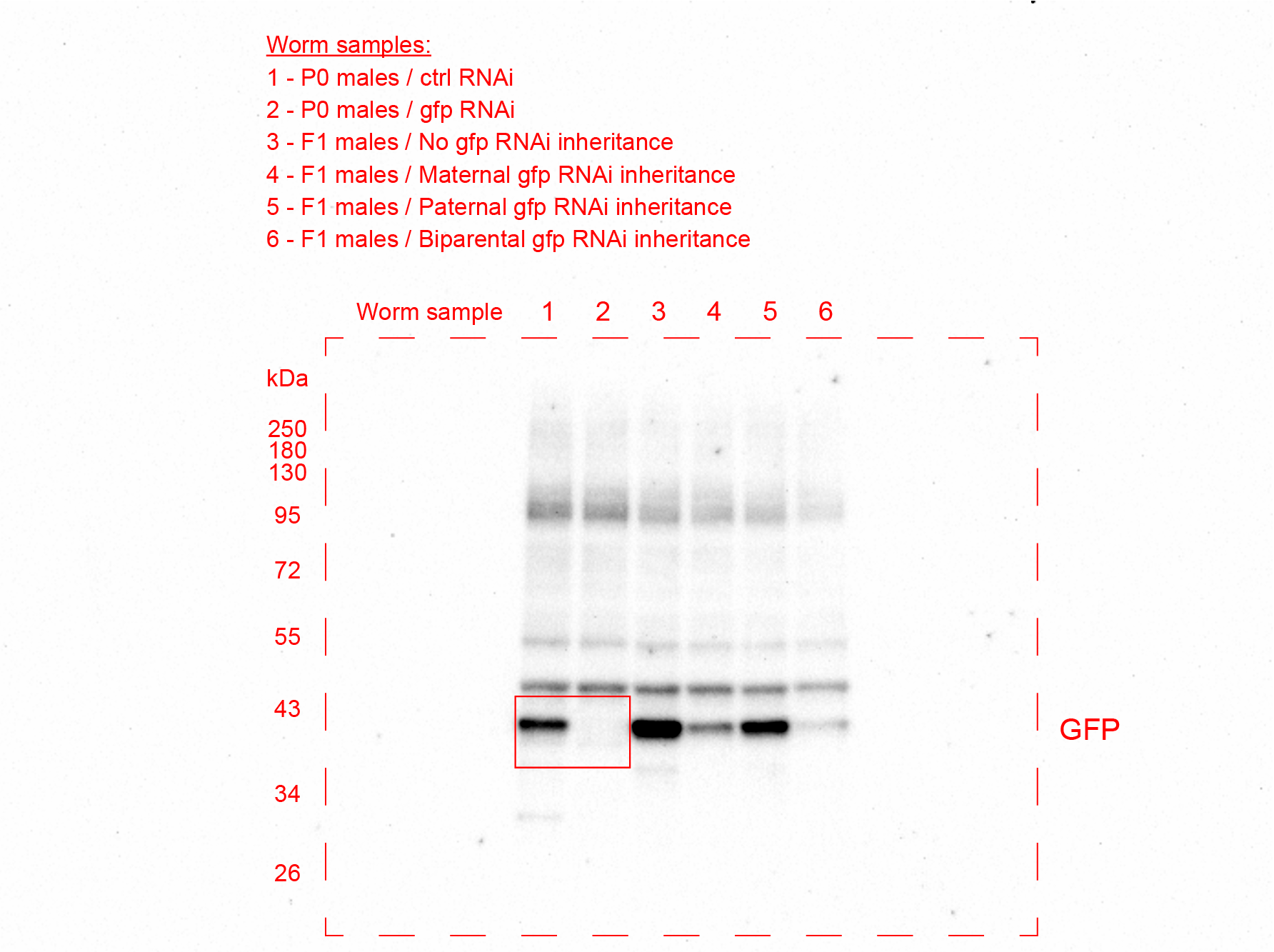

Supplement: Supplementary file 2 — Source data Fig. 1 [file 44319_2025_512_MOESM2_ESM.zip › Figure_1/1D/Western_Blot_anti-GFP_P0_male.png]

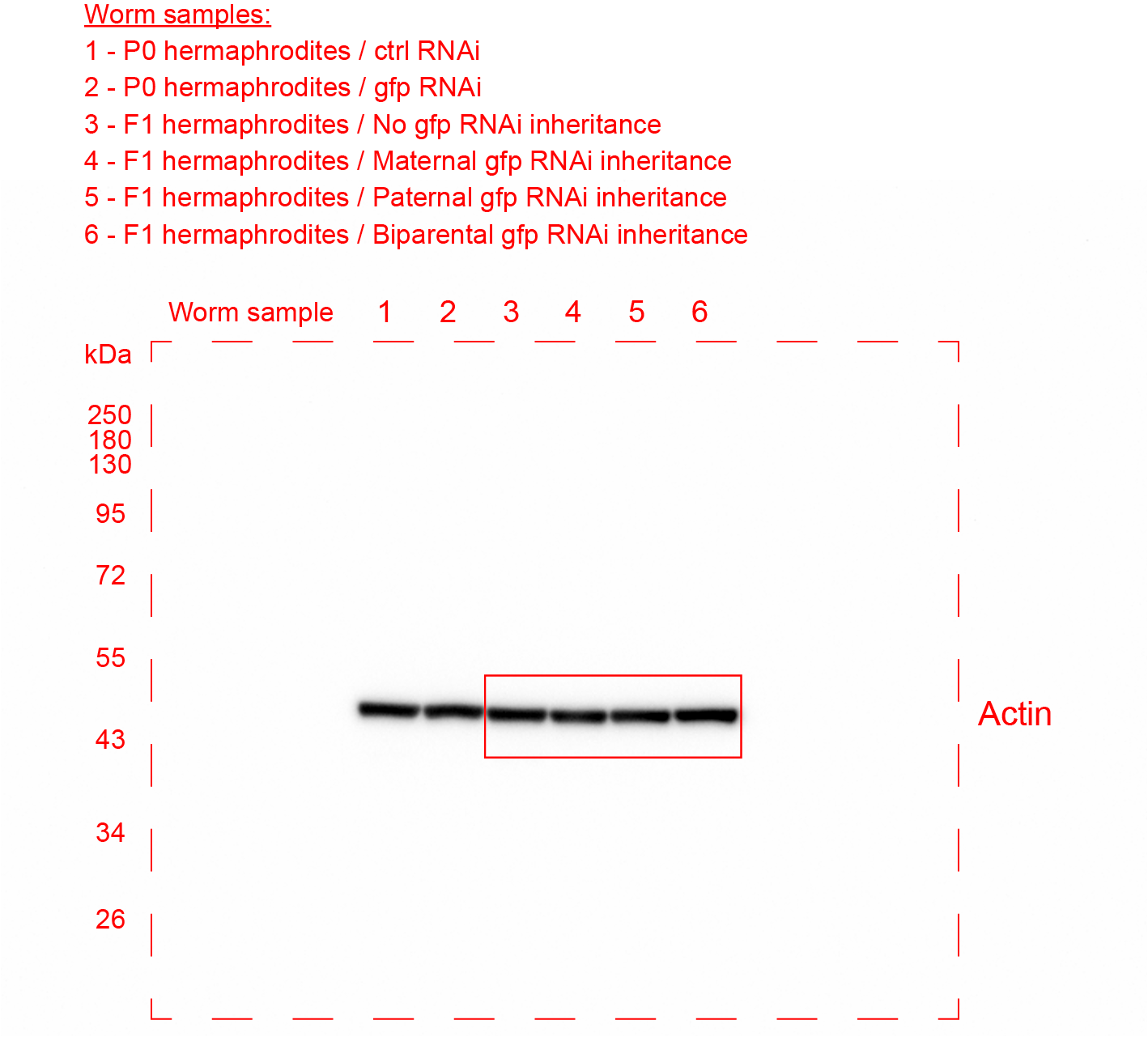

Supplement: Supplementary file 2 — Source data Fig. 1 [file 44319_2025_512_MOESM2_ESM.zip › Figure_1/1J/Western_Blot_anti-actin_F1_hermaphrodite.png]

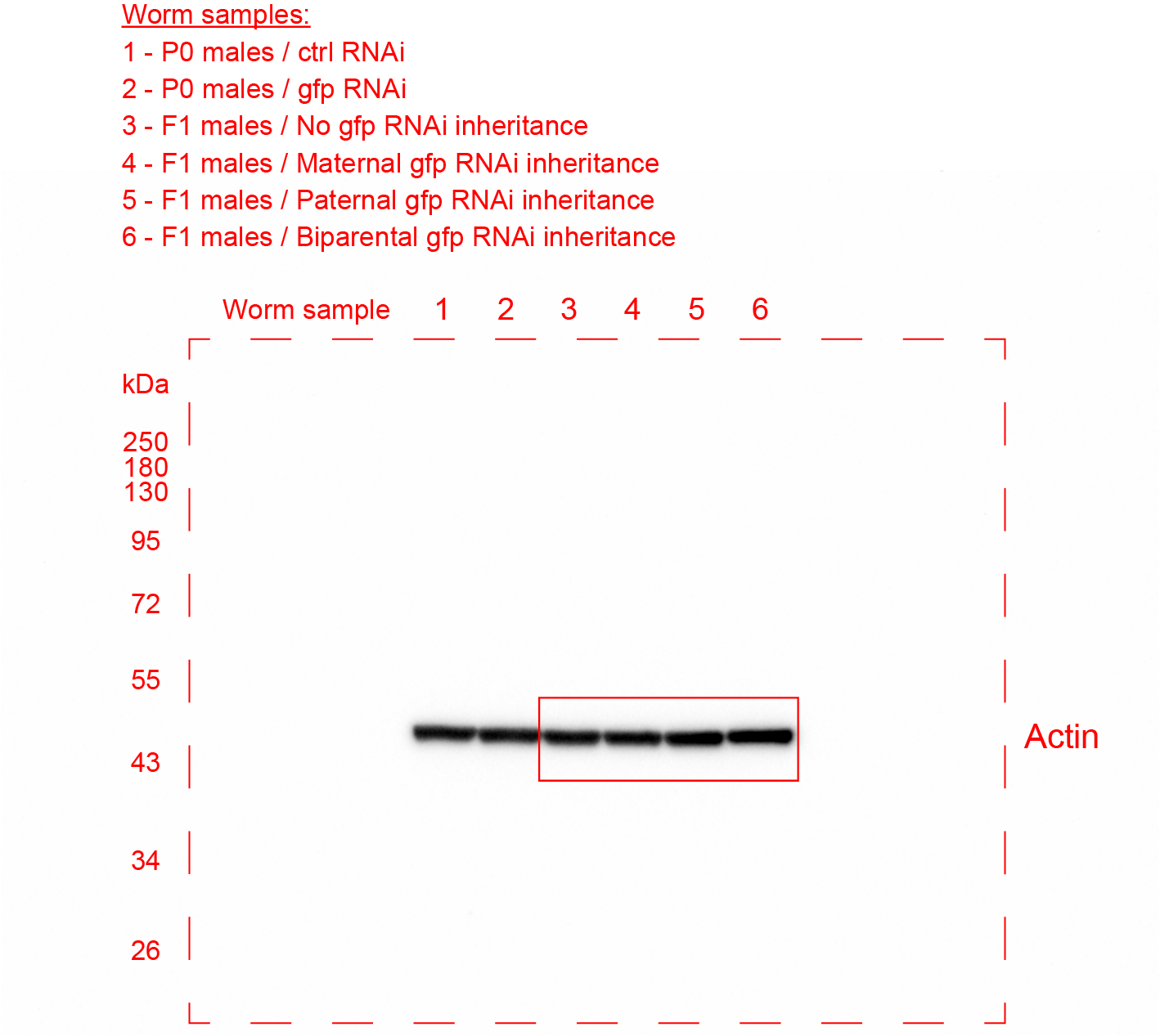

Supplement: Supplementary file 2 — Source data Fig. 1 [file 44319_2025_512_MOESM2_ESM.zip › Figure_1/1J/Western_Blot_anti-actin_F1_male.png]

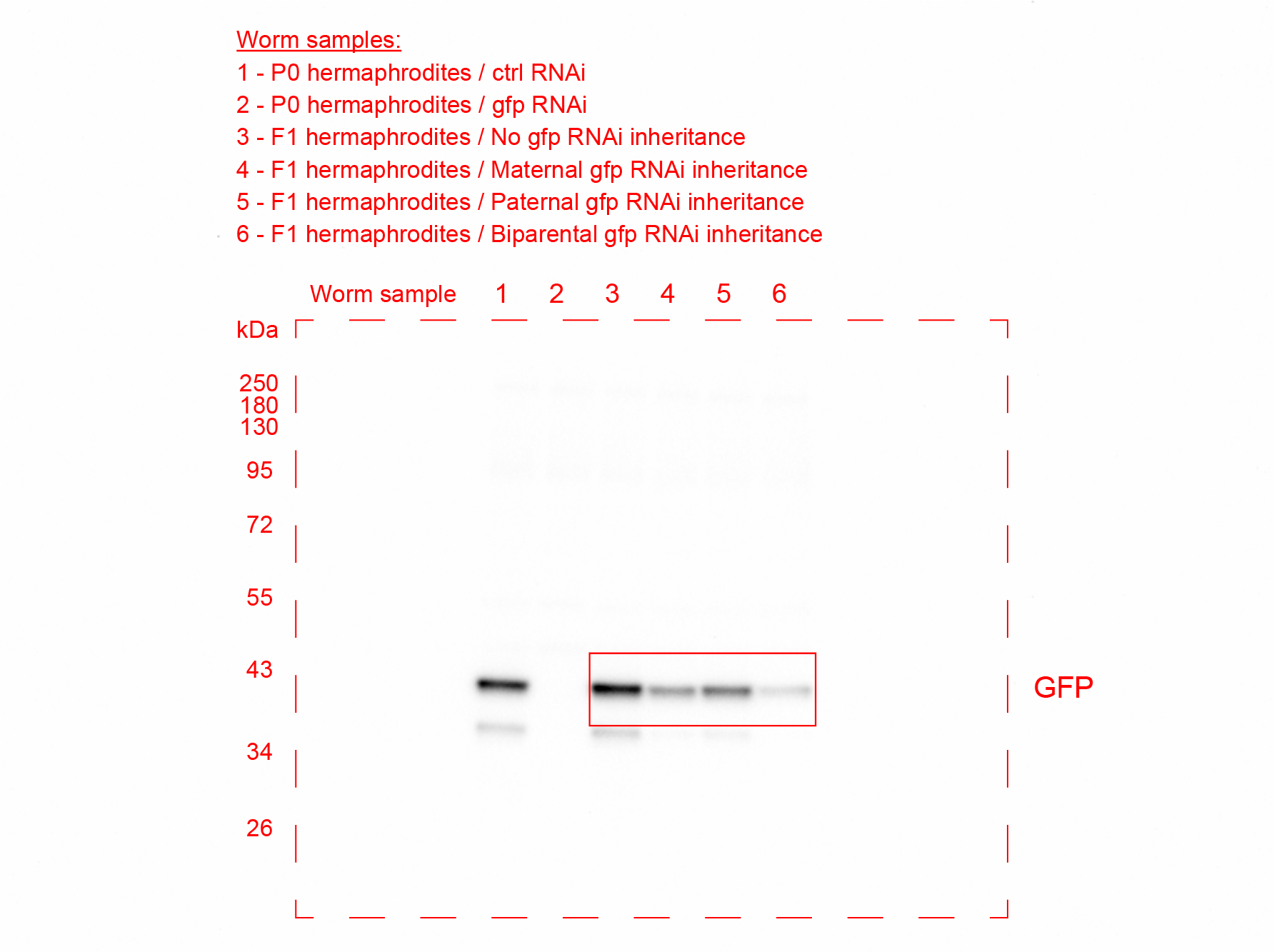

Supplement: Supplementary file 2 — Source data Fig. 1 [file 44319_2025_512_MOESM2_ESM.zip › Figure_1/1J/Western_Blot_anti-GFP_F1_hermaphrodite.png]

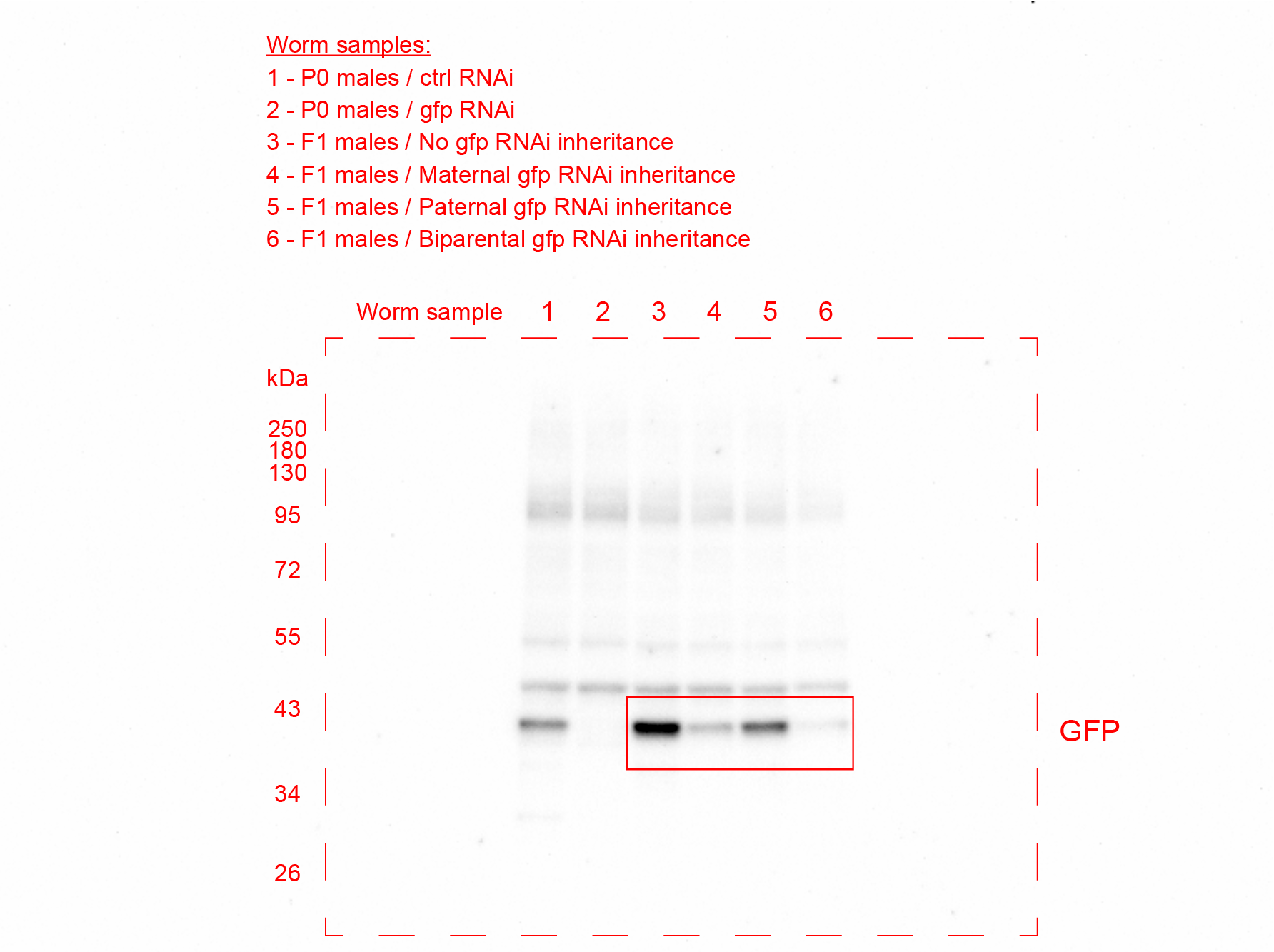

Supplement: Supplementary file 2 — Source data Fig. 1 [file 44319_2025_512_MOESM2_ESM.zip › Figure_1/1J/Western_Blot_anti-GFP_F1_male.png]
