# Supplementary material for: A genetic framework for RNAi inheritance in Caenorhabditis elegans
Source: EMBO Rep. 2025 Jul 7;26(16):4072–99. doi: 10.1038/s44319-025-00512-7 (PMC12373942; doi:10.1038/s44319-025-00512-7)
Supplement: Supplementary file 13 — Expanded View Figures [file 44319_2025_512_MOESM13_ESM.pdf]

Expanded View Figures

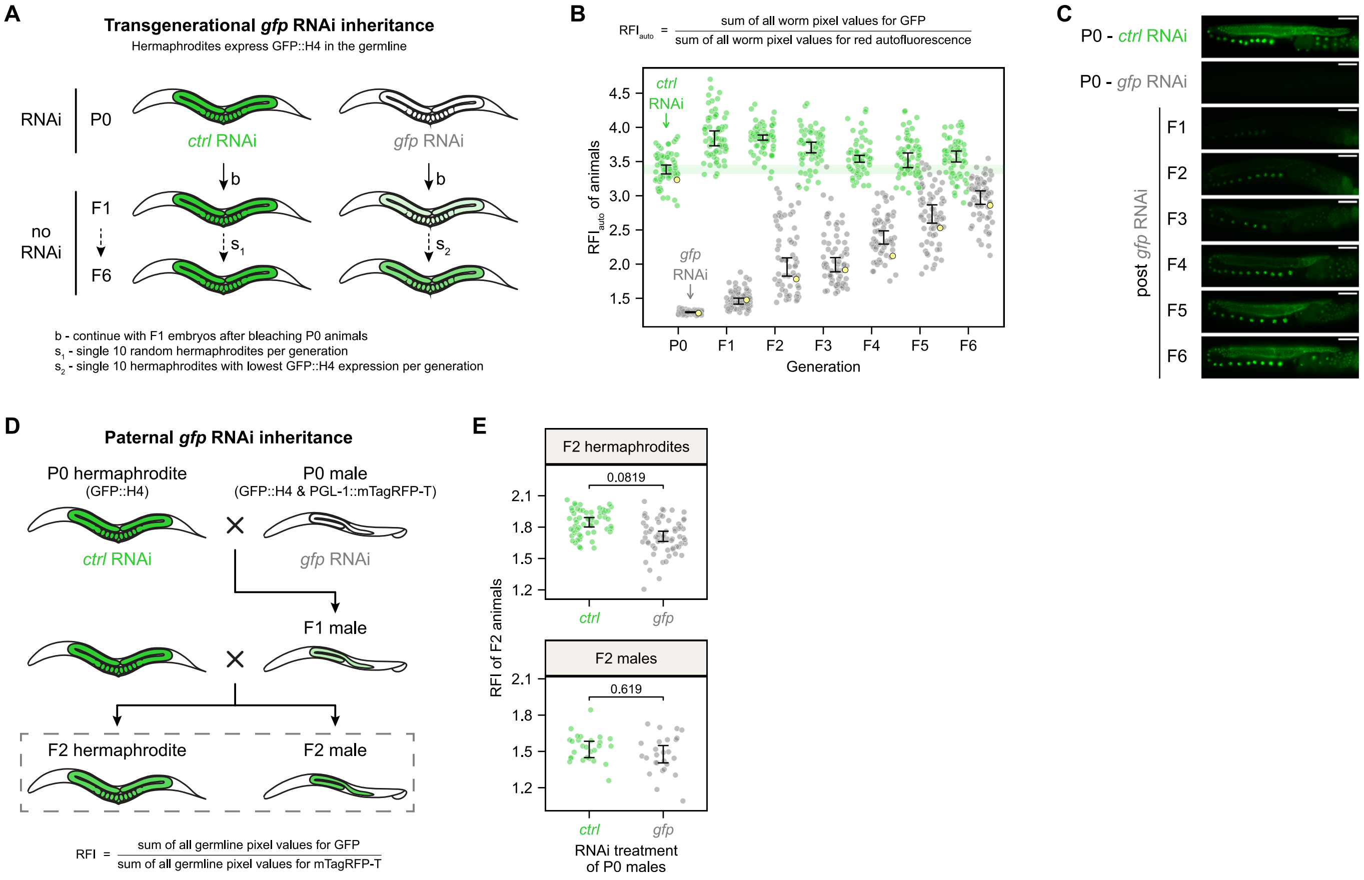

Figure EV1. Transgenerational *gfp* RNAi inheritance of GFP::H4.

(**A**) Schematic representation summarizing the effects of transgenerational *gfp* RNAi inheritance in hermaphrodites expressing GFP::H4 in the germline. Germline color illustrates GFP::H4 expression: green—expressed, shades of light green—expressed at lower levels, white—silenced. (**B**) Relative fluorescence intensity (RFI_{auto}) of GFP::H4 in hermaphrodites before and during transgenerational RNAi inheritance. Only P0 hermaphrodites were treated with either *control* RNAi (green) or *gfp* RNAi (gray). Each dot represents an individual animal, with yellow dots referring to representative micrographs shown in (**C**). 95% confidence intervals of the median are shown as black error bars for all samples as well as green line for the P0 generation treated with *control* RNAi. Sample sizes: P0 = ~60 animals per condition, F1–F6 = ~60 animals from 10 founders per condition. (**C**) Widefield fluorescence micrographs of representative hermaphrodites before and during transgenerational *gfp* RNAi inheritance, as indicated in (**B**). GFP::H4 appears in green. Generations and RNAi conditions are indicated to the left of the micrographs. Scale bars: 50 µm. (**D**) Crossing scheme summarizing the effect of paternal *gfp* RNAi inheritance on germline GFP::H4 expression in F2 animals. Germline color illustrates GFP::H4 expression: green—expressed, light green—expressed at lower level, white—silenced. (**E**) Relative fluorescence intensity (RFI) of GFP::H4 in F2 hermaphrodites and F2 males after paternal RNAi inheritance. P0 males were treated with either *control* RNAi (green) or *gfp* RNAi (gray). P0 hermaphrodites were treated with *control* RNAi. Each dot represents an individual animal. 95% confidence intervals of the median, created with bootstrapping (R = 5000) and the BCa (Bias-Corrected and Accelerated Confidence Intervals) method, are shown as black error bars. Statistically significant differences were determined using two-sided unpaired Wilcoxon rank-sum tests. Sample size = ~60 (hermaphrodite) and ~27 (male) F2 animals from 5 F1 founders and 5 P0 founders per condition. Source data are available online for this figure.

 © The Author(s)

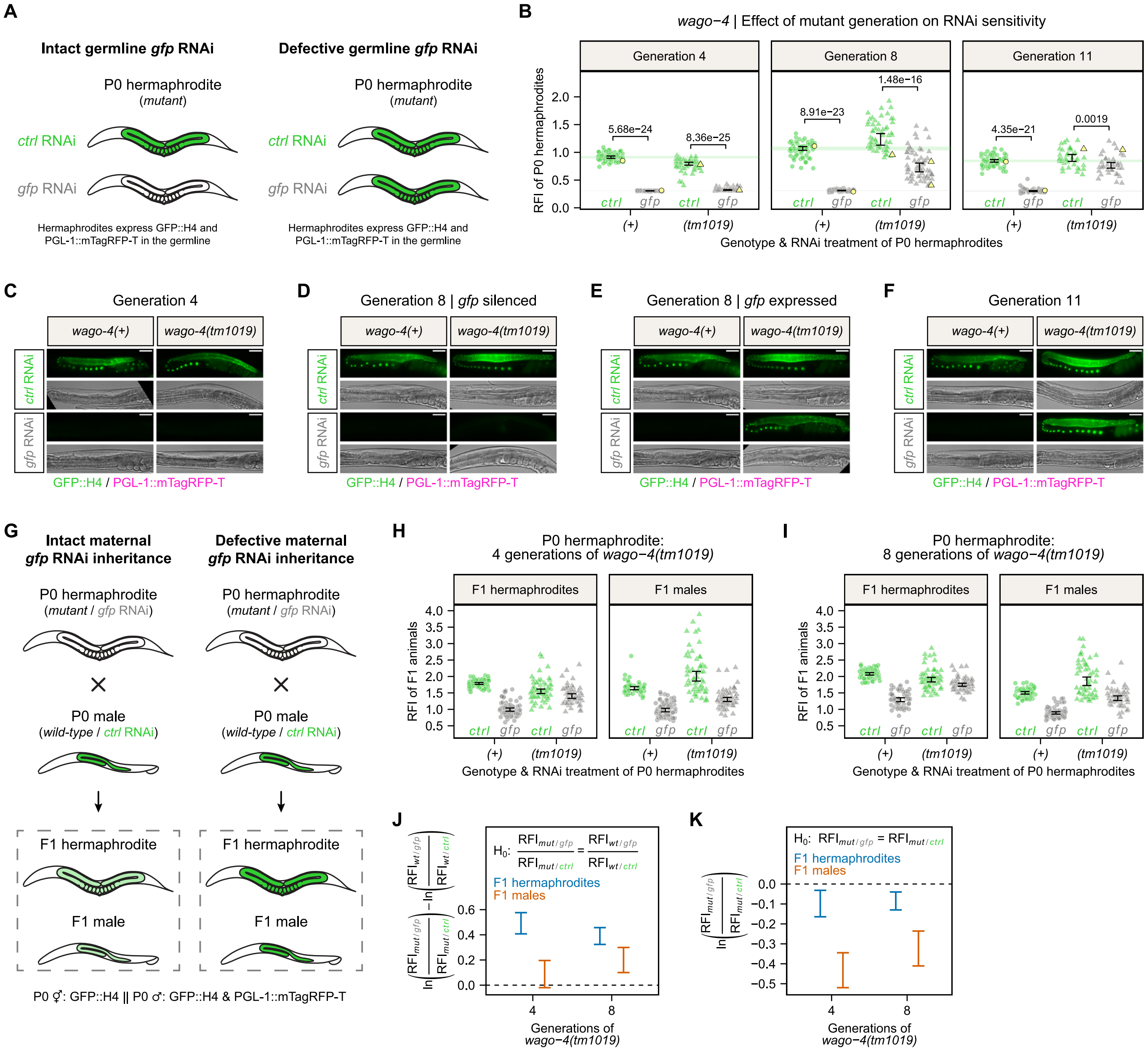
A
Intact germline gfp RNAi
Defective germline gfp RNAi
P0 hermaphrodite (mutant)
ctrl RNAi
gfp RNAi
Hermaphrodites express GFP::H4 and PGL-1::mTagRFP-T in the germline
B
wago−4 | Effect of mutant generation on RNAi sensitivity
Generation 4
Generation 8
Generation 11
RFI of P0 hermaphrodites
5.68e−24
8.36e−25
8.91e−23
1.48e−16
4.35e−21
0.0019
Genotype & RNAi treatment of P0 hermaphrodites
C
Generation 4
D
Generation 8 | gfp silenced
E
Generation 8 | gfp expressed
F
Generation 11
wago-4(+)
wago-4(tm1019)
GFP::H4 / PGL-1::mTagRFP-T
G
Intact maternal gfp RNAi inheritance
Defective maternal gfp RNAi inheritance
P0 hermaphrodite (mutant / gfp RNAi)
P0 male (wild-type / ctrl RNAi)
F1 hermaphrodite
F1 male
P0 ⚥: GFP::H4 || P0 ♂: GFP::H4 & PGL-1::mTagRFP-T
H
P0 hermaphrodite: 4 generations of wago−4(tm1019)
I
P0 hermaphrodite: 8 generations of wago−4(tm1019)
F1 hermaphrodites
F1 males
RFI of F1 animals
Genotype & RNAi treatment of P0 hermaphrodites
J
K
Generations of wago−4(tm1019)

Figure EV2. *wago-4* mutant hermaphrodites become transgenerationally insensitive to germline RNAi.

(**A**) Schematic representations summarizing the *gfp* RNAi effect on mutant P0 hermaphrodites expressing GFP::H4 and PGL-1::mTagRFP-T in the germline. Germline color illustrates GFP::H4 expression: green—expressed, white—silenced. (**B**) Relative fluorescence intensity (RFI) of GFP::H4 in P0 hermaphrodites treated with either *control* RNAi (green) or *gfp* RNAi (gray). P0 hermaphrodites were either wild-type (circle) or mutant (triangle) for *wago-4*, with the latter being analyzed after 4, 8 and 11 generations of mutant homozygosity. Each dot represents an individual animal, with yellow dots referring to representative micrographs shown in (**C–F**). 95% confidence intervals of the median are shown as black error bars for all samples as well as green (*control* RNAi) and gray (*gfp* RNAi) lines for wild-type conditions. Statistically significant differences were determined using two-sided Wilcoxon rank-sum tests. Raw data of 'Generation 8' is identical to Fig. 2E. Sample size = ~60 animals per condition. (exception: ~40 *wago-4(tm1019)* animals for 'Generation 11'). (**C–F**) Widefield fluorescence micrographs of representative P0 hermaphrodites treated with either *control* RNAi or *gfp* RNAi, as indicated in (**B**). P0 hermaphrodites were either wild-type or mutant for *wago-4*, with latter being analyzed after 4 (**C**), 8 (**D**, **E**) and 11 (**F**) generations of mutant homozygosity. GFP::H4 and PGL-1::mTagRFP-T appear in green and magenta, respectively. Micrographs in (**D**) are the same as shown in Fig. 2J. Micrographs in (**E**) representing (i) *wago-4(+)|ctrl* RNAi, (ii) *wago-4(+)|gfp* RNAi, (iii) *wago-4(tm1019)|ctrl* RNAi are the same as shown in Figs. 2J and EV2D. Scale bars: 50 µm. (**G**) Crossing schemes summarizing how mutations in P0 hermaphrodites affect germline GFP::H4 expression in F1 animals after maternal *gfp* RNAi inheritance. Germline color illustrates GFP::H4 expression: green—expressed, light green—expressed at lower level, white—silenced. (**H**, **I**) Relative fluorescence intensity (RFI) of GFP::H4 in F1 hermaphrodites and F1 males after maternal RNAi inheritance. P0 hermaphrodites were treated with either *control* RNAi (green) or *gfp* RNAi (gray), and were either wild-type (circle) or mutant (triangle) for *wago-4*. Mutant P0 hermaphrodites carried the *wago-4(tm1019)* mutation for either 4 (**H**) or 8 (**I**) generations. P0 males were always wild-type for *wago-4* and treated with *control* RNAi. Each dot represents an individual animal. 95% confidence intervals of the mean are shown as black error bars. Raw data of (**I**) is identical to Fig. 3H. Sample size = ~60 F1 animals from 5 P0 founders per condition. (**J**) Comparison of the relative GFP::H4 fluorescence reduction after maternal *gfp* RNAi inheritance between F1 animals sired by *wago-4(+)* and *wago-4(tm1019)* P0 hermaphrodites. The plot summarizes the Gaussian models fitted in (**H**, **I**) and depicts 95% confidence intervals (CIs) of differences of mean log fold changes. The mean differences in log fold changes are in the center of the CIs. The null hypothesis (H_0) expresses equality of relative GFP::H4 fluorescence reduction between wild-type and mutant condition, meaning that the mutation does not cause an enhanced or defective maternal *gfp* RNAi inheritance. A 95% CI not including zero, is equivalent to a rejection of the null hypothesis at the 5% significance level and indicates that the mutation caused a defective (95% CI > 0) maternal *gfp* RNAi inheritance. Color of CIs indicates sex of F1 animals: blue—hermaphrodite, red—male. Sample size = ~60 F1 animals from 5 P0 founders per condition. (**K**) Comparison of the relative GFP::H4 fluorescence intensity after maternal RNAi inheritance between F1 animals sired by *wago-4(tm1019)* P0 hermaphrodites treated with either *control* RNAi or *gfp* RNAi. The plot summarizes the Gaussian models fitted in (**H**, **I**) and depicts 95% confidence intervals (CIs) of mean RFI log fold changes. The mean log fold changes are in the center of the CIs. The null hypothesis (H_0) expresses equality of relative GFP::H4 fluorescence intensity between *control* RNAi and *gfp* RNAi treatments, meaning that the mutation causes a completely defective maternal *gfp* RNAi inheritance. A 95% CI not including zero is equivalent to a rejection of the null hypothesis at the 5% significance level and indicates that the mutation does not cause a completely defective maternal *gfp* RNAi inheritance. Color of CIs indicates sex of F1 animals: blue—hermaphrodite, red—male. Sample size = ~60 F1 animals from 5 P0 founders per condition. Source data are available online for this figure.

© The Author(s)

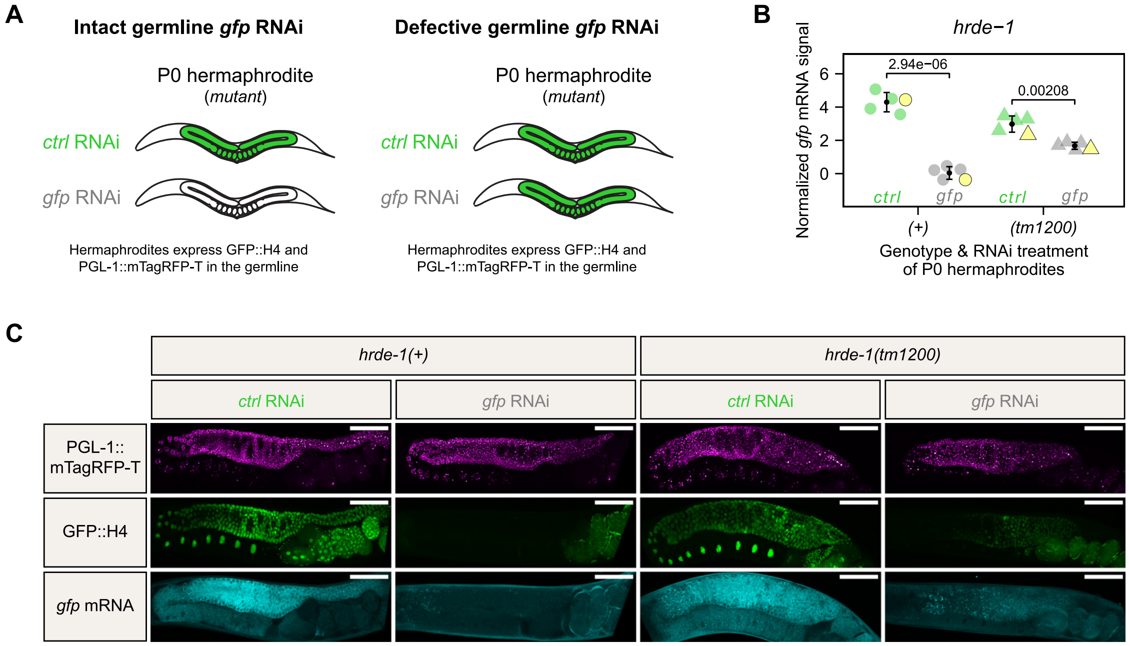

Figure EV3. ***hrde-1* mutant hermaphrodites show impaired RNAi sensitivity.**

(**A**) Schematic representations summarizing the *gfp* RNAi effect on mutant P0 hermaphrodites expressing GFP::H4 and PGL-1::mTagRFP-T in the germline. Germline color illustrates GFP::H4 expression: green—expressed, white— silenced. (**B**) Normalized smFISH signal of *gfp* mRNA in P0 hermaphrodites treated with either *control* RNAi (green) or *gfp* RNAi (gray). P0 hermaphrodites were either wild-type (circle) or mutant (triangle) for *hrde-1*. Each colored dot represents an individual animal, with yellow dots referring to representative micrographs shown in (**C**). The black dot and error bars represent the mean and standard deviation, respectively. *P* values were calculated using an unpaired two-tailed Student's *t* test. Sample size = 5 animals per condition. (**C**) Confocal maximum intensity projections of representative P0 hermaphrodites treated with either *control* RNAi or *gfp* RNAi, as indicated in (**B**). P0 hermaphrodites were either wild-type or mutant for *hrde-1*. GFP::H4, PGL-1::mTagRFP-T and *gfp* mRNA appear in green, magenta and cyan, respectively. Scale bars: 50 µm. Source data are available online for this figure.

© The Author(s)

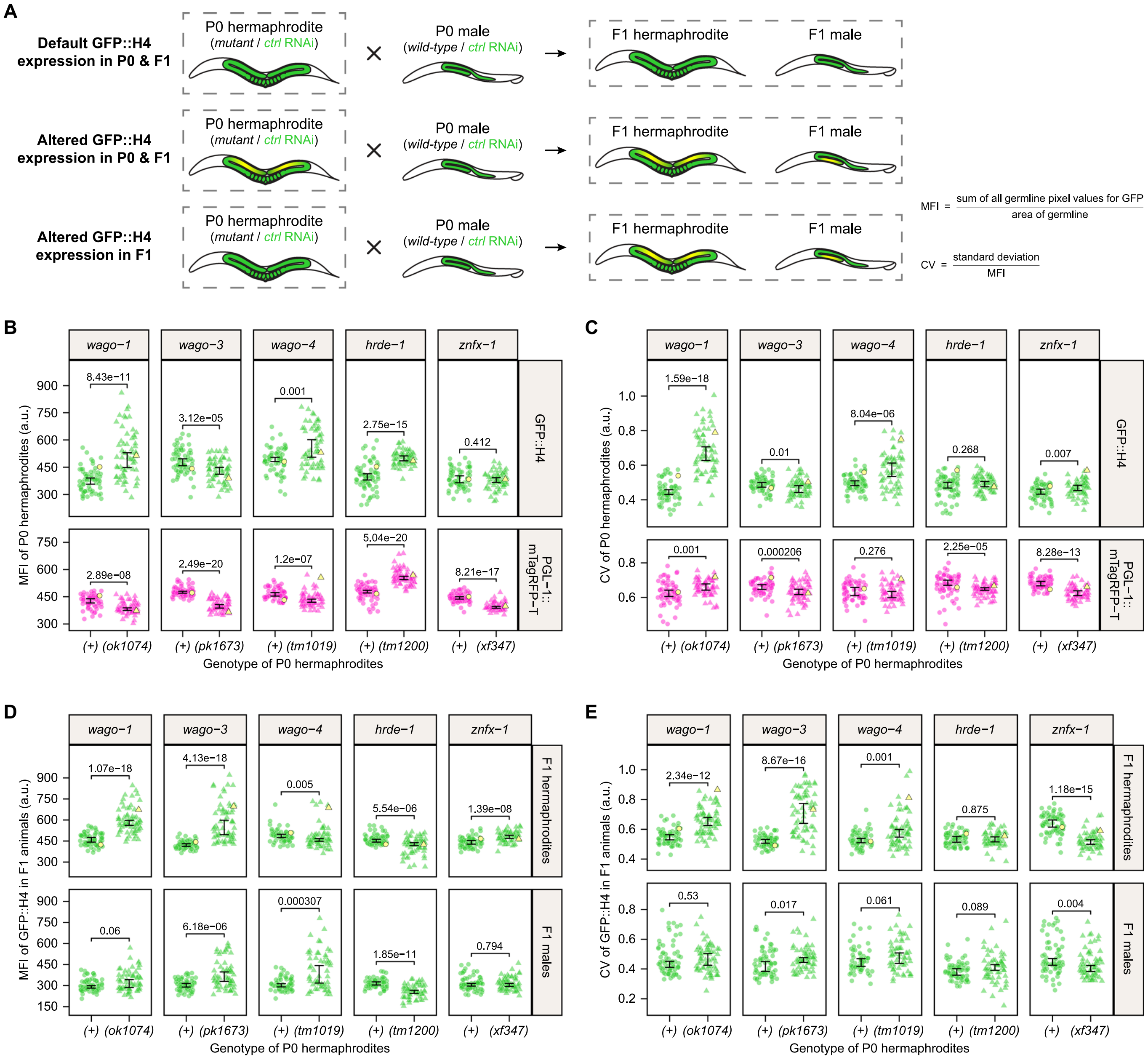

Figure EV4. Global and local effects of mutant RNAi factors on germline GFP::H4 expression in hermaphrodites and their progeny.

(**A**) Crossing schemes summarizing how mutations in P0 hermaphrodites affect germline GFP::H4 expression in F1 animals produced by allogamy. Germline color illustrates GFP::H4 expression: uniformly green—default expression, yellow-green – mutant expression. (**B, C**) Mean fluorescence intensity (MFI) (**B**) and coefficient of variation (CV) (**C**) of GFP::H4 (green) and PGL-1::mTagRFP-T (magenta) in P0 hermaphrodites treated with *control* RNAi. P0 hermaphrodites were either wild-type (circle) or mutant (triangle) for indicated genes. Each dot represents an individual animal, with yellow dots referring to representative animals shown in Fig. 2. 95% confidence intervals of the median are shown as black error bars. Statistically significant differences were determined using two-sided Wilcoxon rank-sum tests. Raw data is identical to Fig. 2. The CV is defined as the ratio of the standard deviation to the mean. Sample size = ~60 animals per condition. (**D, E**) Mean fluorescence intensity (MFI) (**D**) and coefficient of variation (CV) (**E**) of GFP::H4 in F1 hermaphrodites and F1 males after maternal RNAi inheritance. P0 hermaphrodites were treated with *control* RNAi, and were either wild-type (circle) or mutant (triangle) for indicated genes. P0 males were always wild-type for these genes and treated with *control* RNAi. Each dot represents an individual animal, with yellow dots referring to representative animals shown in Fig. 3. 95% confidence intervals of the median are shown as black error bars. Statistically significant differences were determined using two-sided Wilcoxon rank-sum tests. Raw data is identical to Fig. 3. The CV is defined as the ratio of the standard deviation to the mean. Sample size = ~60 F1 animals from 5 P0 founders per condition. Source data are available online for this figure.

 © The Author(s)

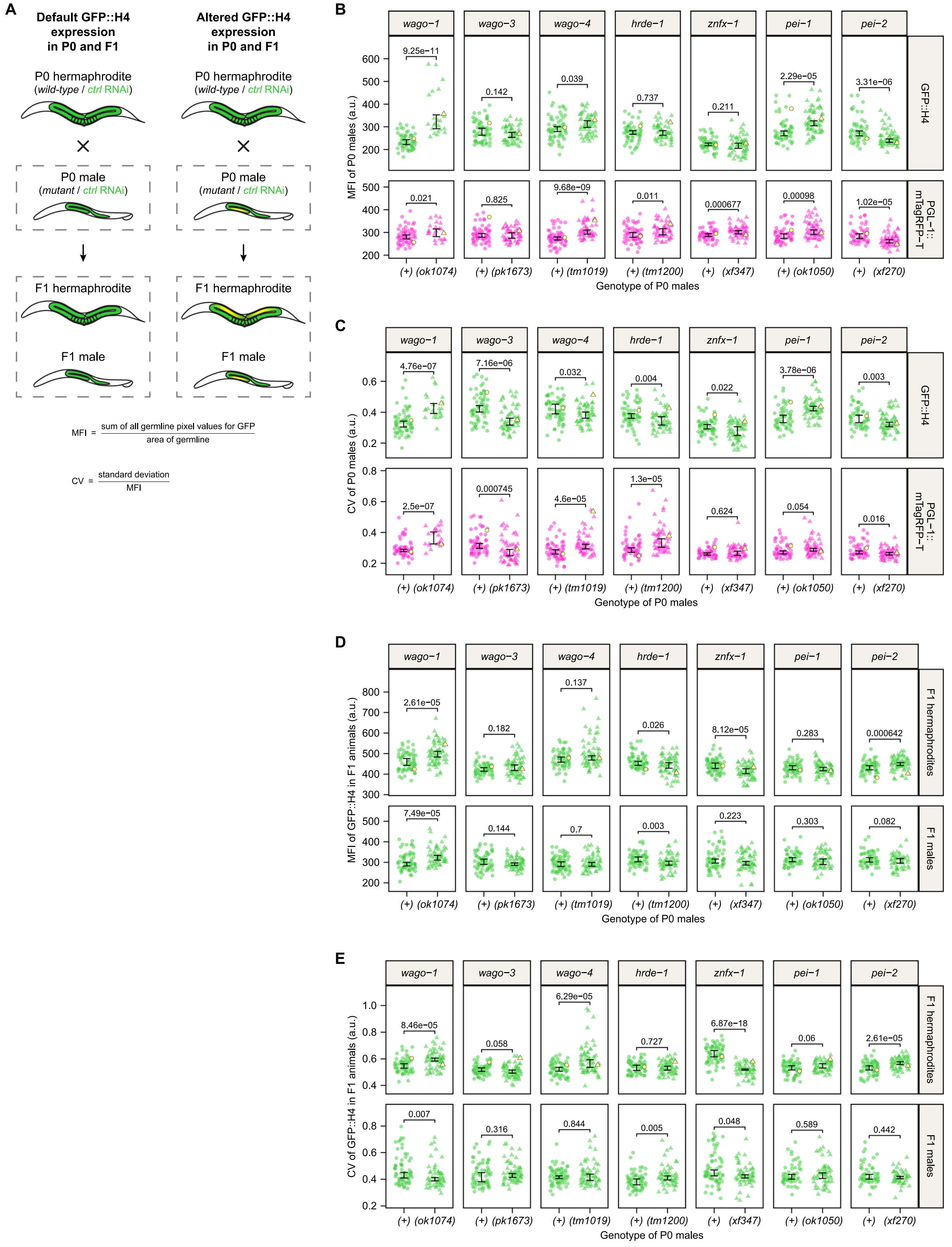
A
Default GFP::H4 expression in P0 and F1
Altered GFP::H4 expression in P0 and F1
P0 hermaphrodite (wild-type / ctrl RNAi)
P0 male (mutant / ctrl RNAi)
F1 hermaphrodite
F1 male
MFI = sum of all germline pixel values for GFP / area of germline
CV = standard deviation / MFI
B
wago−1
wago−3
wago−4
hrde−1
znfx−1
pei−1
pei−2
GFP::H4
PGL−1:: mTagRFP−T
MFI of P0 males (a.u.)
9.25e−11
0.142
0.039
0.737
0.211
2.29e−05
3.31e−06
0.021
0.825
9.68e−09
0.011
0.000677
0.00098
1.02e−05
(+) (ok1074)
(+) (pk1673)
(+) (tm1019)
(+) (tm1200)
(+) (xf347)
(+) (ok1050)
(+) (xf270)
Genotype of P0 males
C
CV of P0 males (a.u.)
4.76e−07
7.16e−06
0.032
0.004
0.022
3.78e−06
0.003
2.5e−07
0.000745
4.6e−05
1.3e−05
0.624
0.054
0.016
D
MFI of GFP::H4 in F1 animals (a.u.)
F1 hermaphrodites
F1 males
2.61e−05
0.182
0.137
0.026
8.12e−05
0.283
0.000642
7.49e−05
0.144
0.7
0.003
0.223
0.303
0.082
E
CV of GFP::H4 in F1 animals (a.u.)
8.46e−05
0.058
6.29e−05
0.727
6.87e−18
0.06
2.61e−05
0.007
0.316
0.844
0.005
0.048
0.589
0.442

Figure EV5. Global and local effects of mutant RNAi factors on germline GFP::H4 expression in males and their progeny.

(**A**) Crossing schemes summarizing how mutations in P0 hermaphrodites affect germline GFP::H4 expression in F1 animals produced by allogamy. Germline color illustrates GFP::H4 expression: uniformly green—default expression, yellow-green—mutant expression. (**B-C**), Mean fluorescence intensity (MFI) (**B**) and coefficient of variation (CV) (**C**) of GFP::H4 (green) and PGL-1::mTagRFP-T (magenta) in P0 males treated with *control* RNAi. P0 males were either wild-type (circle) or mutant (triangle) for indicated genes. Each dot represents an individual animal, with yellow dots referring to representative animals shown in Fig. 4. 95% confidence intervals of the median are shown as black error bars. Statistically significant differences were determined using two-sided Wilcoxon rank-sum tests. Raw data is identical to Fig. 4. The CV is defined as the ratio of the standard deviation to the mean. Sample size = ~60 animals per condition (exception: ~23 *wago-1(ok1074)* animals). (**D**, **E**) Mean fluorescence intensity (MFI) (**D**) and coefficient of variation (CV) (**E**) of GFP::H4 in F1 hermaphrodites and F1 males after paternal RNAi inheritance. P0 males were treated with *control* RNAi, and were either wild-type (circle) or mutant (triangle) for indicated genes. P0 hermaphrodites were always wild-type for these genes and treated with *control* RNAi. Each dot represents an individual animal, with yellow dots referring to representative animals shown in Fig. 5. 95% confidence intervals of the median are shown as black error bars. Statistically significant differences were determined using two-sided Wilcoxon rank-sum tests. Raw data is identical to Fig. 5. The CV is defined as the ratio of the standard deviation to the mean. Sample size = ~60 F1 animals from 5 P0 founders per condition. Source data are available online for this figure.

 © The Author(s)
